# A Decade of Catalytic Progress in 1,4-Dihydropyridines (1,4-DHPs) Synthesis (2016-2024)

**DOI:** 10.2174/0115701794374153250307065611

**Published:** 2025-03-21

**Authors:** Aditi Soni, Monika Sharma, Rajesh K. Singh

**Affiliations:** 1 Department of Pharmaceutical Chemistry, Shivalik College of Pharmacy, Nangal, District Ropar, Punjab, 140124, India;; 2 Research Scholar, IKG Punjab Technical University, Jalandhar, District Kapurthala, Punjab, 144603, India

**Keywords:** 1,4-dihydropyridines, green chemistry, heterogeneous catalyst, multicomponent one-pot synthesis, catalyst-driven synthesis, bioactive compounds

## Abstract

1,4-Dihydropyridines (1,4-DHPs) are highly versatile and bioactive compounds known for their pharmacological properties, including cardiovascular, anticancer, and antioxidant activities. Traditional synthesis methods often involve harsh conditions, such as high temperatures, toxic reagents, and lengthy reaction times, leading to poor yields and environmental concerns. Consequently, there has been a growing focus on developing more sustainable, efficient, and eco-friendly alternatives for their synthesis. Among these, the catalytic one-pot multicomponent reaction (MCR) method has emerged as a promising strategy, offering high efficiency. Catalysts play a crucial role in enhancing reaction efficiency and selectivity, with various systems-metal-based, organocatalysts, polymer-supported catalysts, and enzymatic catalysts-each offering unique advantages. Metal catalysts provide high reactivity and selectivity, organocatalysts are more environmentally benign, polymer-supported catalysts offer improved stability and sustainability, and enzymatic catalysts enable highly specific reactions under mild conditions. However, challenges such as catalyst cost, reusability, scalability, and substrate scope remain. This review examines catalytic strategies for 1,4-DHPs synthesis from 2016 to 2024, highlighting reaction conditions, substrates, and yields. The analysis aims to inspire further exploration of new catalytic methods, expanding the application of 1,4-DHPs in medicinal chemistry.

## 1 INTRODUCTION

**Fig. (1):**
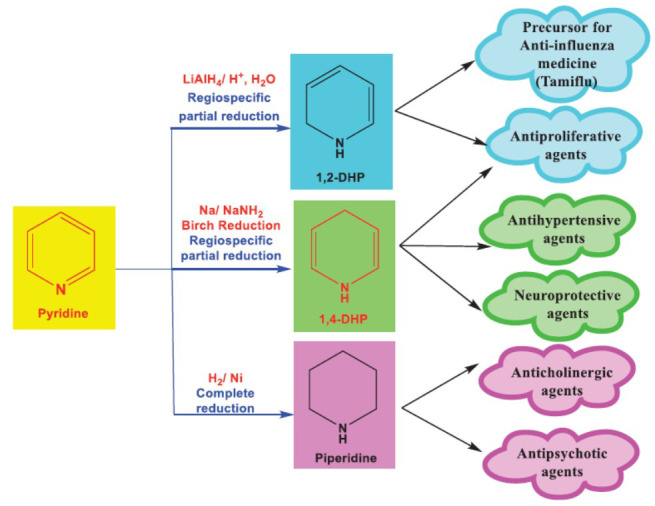
Chemical transformations of pyridine to bioactive scaffolds by various reduction reactions and its pharmacological actions.

Heterocyclic compounds hold a special place in the realm of organic chemistry due to their diverse biological activities and therapeutic applications [1, 2]. Heterocyclic compounds, particularly nitrogen-containing ones, have garnered attention due to their diverse biological activities and pharmacological properties [3, 4]. Pyridine is a six-membered heterocyclic compound containing five carbon atoms and one nitrogen atom and is basic in nature [5]. It is the most commonly found in various products such as plant alkaloids, coffee, rice, bakery products, tea, dairy products, and corn. Anderson first discovered pyridine in bone oil in 1849, and it was also present in coal tar. In 1869, Korner independently discovered the structural relationship between benzene and pyridine, a finding later confirmed by Dewar in 1971. From *Aplopappus harwigi*, pyridine was isolated. In 1876, William Ramsay successfully synthesized pyridine by combining acetylene and hydrogen cyanide in a high-temperature iron furnace. Getting rid of unsaturated double bonds in pyridine rings creates strong scaffolds that can be used in a number of effective pyridine chemical reactions. Pyridine and its reduction products are important in medicinal chemistry due to their diverse biological activities [6]. Researchers have made many scaffolds by partially or completely reducing the number of double bonds in pyridine. This has led to the production of useful 1,2-dihydropyridine (1,2-DHP), 1,4-Dihydropyridine (1,4-DHP), and piperidine (Fig. **1**) [7, 8]. There are only reduction synthesis methods shown in this figure that change pyridine into useful pharmacophores like piperidine, 1,2-DHP, and 1,4-DHP. Piperidine is a saturated heterocyclic amine with a six-membered ring, exhibiting versatile chemistry in pharmaceuticals, agrochemicals, and industrial applications. It has been used as a solvent, adhesive, or chemical intermediate for the synthesis of dyes, pigments, *etc*. [9].

Hantzsch made a significant contribution to medicinal chemistry in 1882 by synthesizing dihydropyridine (DHP), introducing a valuable scaffold for drug development [10]. Among the five potential regioisomers of DHP, the 1,2- and 1,4-DHP forms have garnered the most attention [11]. Notably, several alkaloids, including dioscorine, recognize 1,2-DHPs as precursors for the creation of the isoquinuclidines ring system. Moreover, an isoquinuclidine intermediate, derived from 1,2-DHP, plays a crucial role in synthesizing the anti-influenza medication oseltamivir phosphate (Tamiflu). As a result, 1,2-DHP scaffolds serve as key intermediates or precursors in pharmaceutical synthesis. However, their uses are more limited compared to the broader range of applications for 1,4-DHP compounds [11].

Among these scaffolds, 1,4-DHPs are the most abundant and significant. Numerous natural and synthetic compounds incorporate 1,4-DHPs, which are associated with molecules exhibiting remarkable pharmacological properties [12]. The basicity of 1,4-DHP stems from the lone pair of electrons on the nitrogen atom, making it highly reactive with electrophilic reagents. Naturally occurring derivatives, such as nicotinamide adenine dinucleotide (NADH) and nicotinamide adenine dinucleotide phosphate (NADPH), underscore the importance of 1,4-DHPs in medicinal chemistry and therapeutic applications. 1,4-DHPs display diverse pharmacological activities, including antihypertensive, neuroprotective, antimicrobial, vasodilatory, anti-inflammatory, and antidiabetic effects [13]. The broad significance of 1,4-DHPs has therefore driven organic chemists to design these compounds in simple and efficient ways.

Hantzsch's pioneering work in synthesizing 1,4-DHPs involved reacting aldehydes with two moles of ethyl acetoacetate and ammonia in a one-pot multicomponent reaction (MCR), laying the foundation for subsequent developments in 1,4-DHP synthesis [10]. The mechanism of this one-pot MCR, known as the Hantzsch synthesis, is depicted in Fig. (**2**). Initially, an aldehyde (R-CHO) undergoes condensation with one equivalent of a β-keto ester (OR^2^) to yield intermediate-I. A second β-keto ester reacts with ammonia to form intermediate-II. Sequential condensation steps remove water molecules, leading to the formation of intermediates I and II. These intermediates then cyclize through tautomerization, forming a dihydropyridine core structure (III to IV). This step involves the elimination of ammonium (NH_4_^+^) and ammonia (NH_3_), which drives the reaction forward. Further rearrangements stabilize the cyclic structure, and the final product is a 1,4-DHP derivative. The substituents (R, R^1^, and OR^2^) attached to the core structure dictate the chemical and pharmacological properties of the compound [14].

Over the years, researchers have refined the classical methodology for synthesizing 1,4-DHPs by exploring various catalytic systems that optimize reaction conditions and improve product selectivity. Despite these advancements, previous studies have identified several limitations in the existing catalytic systems. Continuous exploration of novel catalysts is essential to overcoming these challenges. The primary objective is to enhance catalytic efficiency, improve sustainability, expand substrate scope, and develop cost-effective catalysts that can accelerate the development of new pharmaceuticals and therapeutic agents.

However, current catalyst types, such as Lewis acid catalysts, have several drawbacks, including lack of recyclability, sensitivity to moisture, side reactions, and corrosive properties, which limit their practical application [15]. Homogeneous catalysts also face challenges such as thermal instability, difficulty in separation and recovery, and environmental hazards due to the toxicity of certain metal complexes [16]. While heterogeneous catalysts are widely used, they are not without issues, including low selectivity, limited availability of active sites, deactivation over time (*e.g*., fouling or sintering), and reproducibility problems in catalyst preparation [17]. Additionally, common issues across various catalyst systems include limited catalytic efficiency, low yields, poor regioselectivity and stereoselectivity, and non-green reaction conditions. Addressing these challenges through the development of novel catalytic systems is crucial for advancing sustainable and efficient chemical synthesis.

One of the most pressing challenges in modern organic chemistry remains the development of effective and long-lasting methods for synthesizing complex heterocyclic compounds. To address these challenges, catalyst-driven MCRs have emerged as an effective approach for synthesizing 1,4-DHPs [18]. These reactions allow for the simultaneous combination of multiple reactants to form complex products in a single step, significantly improving yields and reducing waste. Recent advancements in this field have led to the development of novel catalyst systems that enhance reaction efficiency while adhering to green chemistry principles [19].

Despite the growing interest in sustainable and green chemistry, which has led to numerous review articles [19-22], there remains a lack of comprehensive reviews focused on the application of green chemistry principles in the catalytic synthesis of 1,4-DHPs. Singh *et al*. limited their review of catalytic synthetic methods for 1,4-DHP to studies published before 2015 [20]. A more recent review by Faizan *et al.* (2024) did not classify the catalysts but instead focused on the catalyst-driven Hantzsch reaction for 1,4-DHP synthesis over the past few years [21]. Similarly, Lavanya *et al.* (2024) discussed only nanomaterial-driven catalytic reactions for synthesizing 1,4-DHPs [22]. However, none of these reviews have provided a thorough examination of the full scope of 1,4-DHP synthesis.

This study aims to address these gaps by offering a comprehensive update on the synthesis of 1,4-DHP from 2016 to the present. The detailed information provided on 1,4-DHP synthesis will highlight benefits such as high yields, scalability, environmental friendliness, and excellent regioselectivity-factors that are especially valuable to new organic chemists working in this field.

## 2 SYNTHETIC APPROACHES

Various synthetic approaches have been adopted for the synthesis of 1,4-DHP, including microwave irradiation, ultrasound-assisted methods, green synthesis, and multicomponent reactions (MCRs), among others. The journey to optimize 1,4-DHP synthesis began with the conventional thermal method, where researchers heated reactants to high temperatures (80-120°C) for extended periods (4-24 hours), which resulted in low yields and excessive energy consumption. To address these limitations, researchers turned to microwave-assisted synthesis (MAO). This innovative technique employed controlled temperatures and short reaction times (1-30 minutes), yielding improved productivity and reduced side reactions [23, 24].

Next, ultrasound-assisted synthesis (UAS) emerged, leveraging sonic waves to enhance reaction rates and minimize energy consumption. This eco-friendly approach reduced reaction times to mere minutes, paving the way for further advancements [25]. The introduction of heterogeneous catalysts marked another significant milestone. Solid catalysts like silica and alumina facilitated mild reaction conditions (50-100°C) and allowed for the reuse of catalysts, reducing waste and costs [26]. Simultaneously, homogeneous catalysts utilizing transition metal complexes and Lewis acids offered high yields and selectivity [15]. As the focus shifted toward sustainability, green chemistry approaches gained prominence. Solvent-free synthesis, water-based reactions, and ionic liquid-based methods minimized environmental impact [27, 28].

MCR simplified the process by integrating multiple reactants in a single pot, reducing waste. The challenge in MCRs lies in preventing the formation of byproducts while achieving high yields of the desired product. The formation of the product depends on various factors, including the type of solvent, temperature variations, catalyst loading, concentration, the nature of the starting materials, and the functional groups involved [29, 30]. Today, chemists can choose from an array of optimized methods, balancing efficiency, selectivity, and sustainability. The latest advancements include the use of nano-catalysts for enhanced reactivity and continuous flow synthesis for improved scalability. These innovations pave the way for the next generation of 1,4-DHP synthesis, promising even greater efficiency, sustainability, and therapeutic potential.

**Scheme 1:**
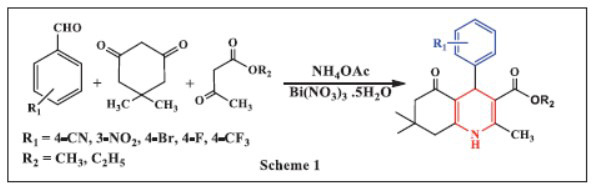
Multi-component one-pot synthesis of 1,4 dihydropyridines using Bismuth nitrate catalyst.

Fig. (**3**) illustrates the different catalysts used to synthesize 1,4-DHP derivatives from multiple components in a single pot. If two moles of the same type of 1,3-diketone are used, the reaction yields a symmetrical product, as shown in Fig. (**3**). Conversely, if one mole of each of two different 1,3-diketones is used, the reaction yields an asymmetrical product, as discussed in Scheme **1**.

## 3 SYNTHESIS OF 1,4-DHP AND ITS DERIVATIVES

Arthur Hantzsch's pioneering work in 1882 marked a significant milestone in the synthesis of dihydropyridines that have since become valuable building blocks for synthesizing novel drugs. Initially, 1,4-DHPs were synthesized by refluxing aldehydes and two moles of ethyl acetoacetate with ammonia [31]. Over the years, these methodologies have been refined, with researchers exploring various catalytic systems to optimize reaction conditions and improve product selectivity [32]. This review analyzes and summarizes the evolution of 1,4-DHP synthesis from 2016 to the present.

In 2016, Mansoor *et al.* described an efficient synthesis of 1,4-DHPs *via* the Hantzsch reaction, catalyzed by bismuth nitrate in four-component coupling reactions of aromatic aldehydes. Several 1,4-DHP derivatives were synthesized effectively by reacting various aryl aldehydes, β-keto compounds such as 5,5-dimethyl-1,3-cyclohexanedione (dimedone), alkyl acetoacetate, and ammonium acetate with a catalytic amount of Bi(NO_3_)_3_·5H_2_O at 75-80°C. Thin layer chromatography (TLC) was employed to monitor the reaction progress. Notably, Bi(NO_3_)_3_ was found to be more effective than other Lewis acids in the synthesis of 1,4-DHP derivatives. This method is appealing due to its mild reaction conditions, ease of experimentation, compatibility with various functional groups, high yields, reduced reaction times, and simple workup procedures. Furthermore, Bi(NO_3_)_3_ offers the added advantage of being recoverable and reusable, making the process more economically viable while enhancing selectivity (Scheme **1**) [33].

**Scheme 2:**
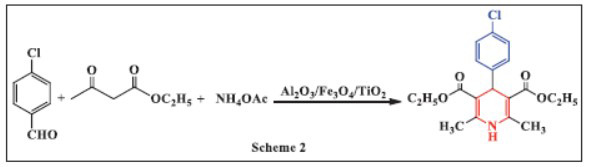
Multi-component one-pot synthesis of 1,4 dihydropyridines using aluminium oxide/iron oxide/titanium dioxide catalysts.

Nakhaei *et al.,* in 2016, demonstrated the catalytic impact of three nano-sized metal oxides-Al_2_O_3_, Fe_3_O_4_, and TiO_2_ nanoparticles-on the synthesis of 1,4-DHPs under various reaction conditions. The one-pot, three-component reaction involved aliphatic or aromatic aldehydes, ammonium acetate, and ethyl acetoacetate in the presence of these catalysts. The reaction yielded 1,4-DHPs effectively, and the impact of temperature on the reaction was also studied. At 80°C, excellent product yields were achieved, and it was observed that the yield increased as the reaction temperature rose. The results revealed that, under thermal, solvent-free conditions, nano-TiO_2_ acted as a more effective heterogeneous catalyst compared to the other catalysts. The reaction proceeded more smoothly and produced the highest yields in shorter reaction times. Advantages of this method include short reaction durations, easy product isolation, and the use of environmentally benign catalysts. Additionally, the catalysts were easily recoverable and reusable in subsequent catalytic cycles (Scheme **2**) [34].

**Scheme 3:**
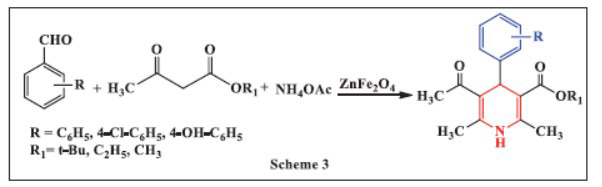
Multi-component one-pot synthesis of 1,4 dihydropyridines using zinc ferrite catalyst.

**Ravikumar *et al.,*** in **2016**, demonstrated an efficient multicomponent synthesis of 1,4-DHPs using a combination of aldehydes, ethyl acetoacetate, and ammonium acetate in water, catalyzed by ZnFe_2_O_4_ nanoparticles. This dual Lewis acid-base catalyst showcases the advantages of using ZnFe_2_O_4_ nanoparticles, which can be magnetically recovered and reused, making the process both economical and environmentally friendly. The use of water as a solvent further enhances the sustainability of the reaction. The method is characterized by high yields and rapid reaction times, significantly outperforming traditional catalysts. The synthesis occurs at room temperature, which not only simplifies the procedure but also minimizes energy consumption. The process is particularly notable for its minimal environmental impact, aligning with contemporary green chemistry principles. By utilizing ZnFe_2_O_4_ nanoparticles, the researchers achieved a notable reduction in reaction time while maintaining high product yields, thus providing a robust alternative for synthesizing 1,4-DHPs (Scheme **3**) [35].

**Scheme 4:**
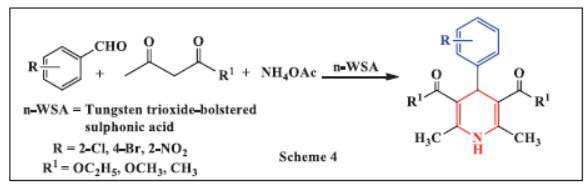
Multi-component one-pot synthesis of 1,4 dihydropyridines using Tungsten trioxide-bolstered sulphonic acid catalyst.

Bitaraf *et al.,* in 2016 reported the synthesis of 1,4-dihydropyridines using tungsten trioxide-supported sulphonic acid (n-WSA) nanoparticles as a catalyst. This catalytic system facilitated the three-component reaction of aromatic aldehydes, β-carbonyl compounds, and ammonium acetate under solvent-free conditions. This technique is economically significant since it requires fewer tests to assess the effects of different parameters on the response. The primary advantage of this approach is its simplicity in purifying, as it only requires simple recrystallization as opposed to column chromatography. This is likely because the resultant compounds are highly pure. The key advantages of newly developed methods include quick reaction times, ease of purification, and an environmentally friendly approach. This synthetic catalyst n-WSA produces resultant product with excellent yields (Scheme **4**) [36].

**Scheme 5:**
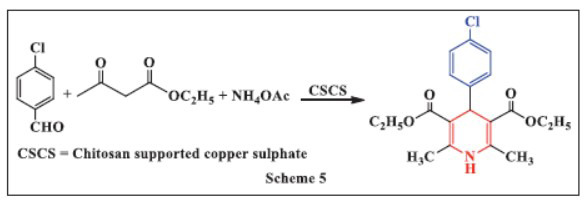
Multi-component one-pot synthesis of 1,4 dihydropyridines using Chitosan supported copper sulphate catalyst.

**Dekamini *et al.,*** in **2016,** developed an efficient method for the synthesis of 1,4-DHPs using chitosan-supported copper (II) sulfate (CSCS) as a catalyst. The reaction mixture was refluxed by simply adding ammonium acetate, β-dicarbonyl compounds, and various aldehydes to a low loading of CSCS in ethanol, yielding 1,4-DHPs. Key advantages of this approach include low catalyst loading, clean reaction profiles, and the use of a one-pot, multicomponent procedure to synthesize medicinally active 1,4-DHP derivatives. Further benefits include the catalyst's reusability, biodegradability, and operational simplicity (Scheme **5**) [37].

**Scheme 6:**
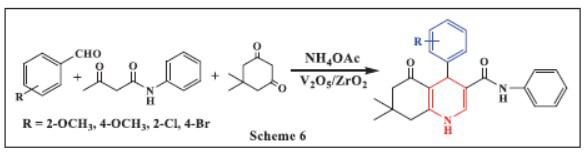
Multi-component one-pot synthesis of 1,4 dihydropyridines using Sulphated polyborate catalyst.

**Bhaskaruni *et al.,*** in **2017,** reported the one-pot synthesis of 1,4-DHPs using multiple components, which involves the cyclo-condensation of aromatic aldehydes, aceto-acetanilide, ammonium acetate, and 5,5-dimethyl-1,3-cyclohexanedione. At room temperature and within a reaction time of less than 20 minutes, eight new 1,4-DHPs were synthesized using ethanol as the solvent and V_2_O_5_/ZrO_2_ as a heterogeneous catalyst. Key advantages of this method include easy work-up, the use of a green solvent, rapid reaction times, mild reaction conditions, and excellent yields (90-96%). X-ray diffraction analysis was employed to examine the phase and crystallinity of the synthesized catalyst. The average crystallite size of V_2_O_5_/ZrO_2_, calculated using the Scherrer equation from the maximum intensity diffraction peak, was approximately 7.6 nm. The reaction product could be readily separated in its pure form without the need for chromatographic separation (Scheme **6**) [38].

**Scheme 7:**
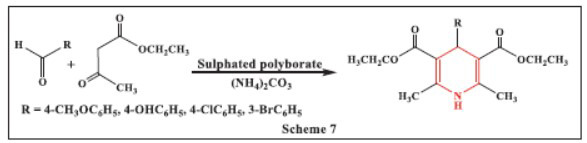
Multi-component one-pot synthesis of 1,4 dihydropyridines using Sulphated polyborate catalyst.

**Rekunge *et al.,*** in **2017,** introduced a simple and efficient method for synthesizing four-component 1,4-dihydropyridines (1,4-DHPs) from various aldehydes, β-ketoesters, and ammonium carbonate. The process utilizes sulphated polyborate as a catalyst, operating at 90 °C without solvents and yielding high results. Notably, the catalyst can be recycled up to four times without significant loss of activity. The key advantages of this method include high yields, rapid reaction times, solvent-free conditions, easy workup, catalyst recyclability, and tolerance to a broad range of functional groups (Scheme **7**) [39].

**Scheme 8:**
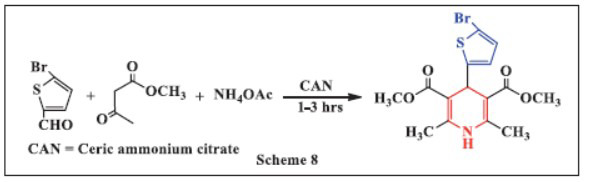
Multi-component one-pot synthesis of 1,4 dihydropyridines using Ceric ammonium nitrate catalyst.

**Sharma *et al.,*** in **2017,** reported a method for synthesizing 1,4-dihydropyridines (1,4-DHPs) using ceric ammonium nitrate (CAN) as a catalyst. This approach involves the reaction of various 1,3-diones, 5-bromothiophene-2-carboxaldehyde, and ammonium acetate at room temperature under solvent-free conditions, significantly reducing reaction time. The progress of the reaction was monitored by TLC. Compared to conventional methods, this solvent-free process proved to be more efficient. The synthesis of Hantzsch dihydropyridine offers several advantages, including shorter reaction times, simplicity, high yields, ease of execution, and cost-effectiveness, making CAN an ideal catalyst for chemical synthesis (Scheme **8**) [40].

**Scheme 9:**
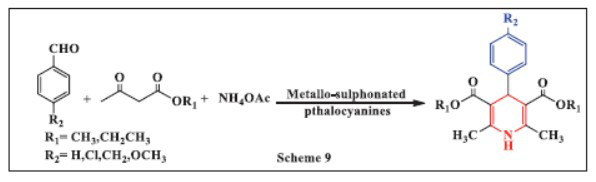
Multi-component one-pot synthesis of 1,4 dihydropyridines using Metallo-sulphonated phthalocyanines catalyst.

**Laura *et al.,*** in **2017,** investigated alternative catalysts, specifically metallo-sulphonated phthalocyanines (FePcS-NH_2_-SiO_2_ and CoPcS-NH_2_-SiO_2_), for a green, multicomponent Hantzsch reaction between aromatic aldehydes, methyl or ethyl acetoacetate, and ammonium acetate. The reaction conditions influenced the product formation, yielding either 1,4-DHPs or phenylpyridines. At room temperature, high selectivity for 2-phenylpyridines was achieved in the absence of oxidants and solvents. In contrast, temperatures above 45°C favored the production of 1,4-DHP derivatives. The FePcS-NH_2_-SiO_2_ catalyst demonstrated excellent stability, maintaining its activity for at least six cycles in the aromatization reaction. Additionally, 1,4-DHP derivatives were successfully aromatized to the corresponding pyridines at 50°C using acetonitrile as the solvent and t-BuOOH as the oxidant (Scheme **9**) [41].

**Scheme 10:**
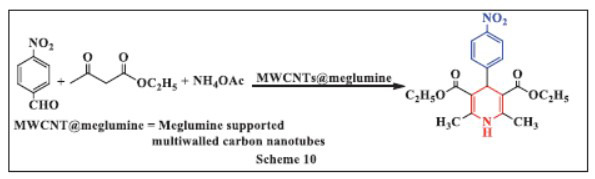
Multi-component one-pot synthesis of 1,4 dihydropyridines using Meglumine supported multiwalled carbon nanotubes catalyst.

**Zare *et al.,*** in **2018,** developed an ultrasound-promoted green synthesis method for 1,4-DHPs. This innovative multi-component synthesis involved using meglumine supported on multiwalled carbon nanotubes (MWCNTs@meglumine) as a highly efficient and reusable heterogeneous catalyst. The reaction combined aryl aldehydes, ammonium acetate, and ethyl acetoacetate in ethanol, with MWCNTs @meglumine catalyzing the process. The progress of the reaction was monitored by thin-layer chromatography, and the reaction was carried out under ultrasonic irradiation at room temperature. This method offers several advantages, including an efficient, reusable catalyst, simple experimental procedures, rapid reaction times, and quick product workup. Furthermore, this highly effective basic catalyst can be extended to the synthesis of other organic molecules (Scheme **10**) [42].

**Scheme 11:**
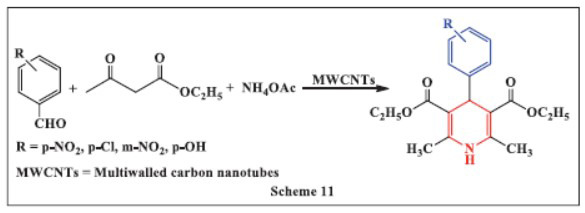
Multi-component one-pot synthesis of 1,4 dihydropyridines using Multiwalled carbon nanotubes catalyst.

**Mahinpour *et al.,*** in **2018,** reported the synthesis of 1,4-DHPs using aminated multiwalled carbon nanotubes (MWCNTs) as a highly efficient catalyst. The method involved stirring a mixture of aryl aldehyde, ammonium acetate, ethyl acetoacetate, and aminated carbon nanotubes in ethanol for an appropriate duration. TLC was used to monitor the reaction progress. The multicomponent reaction yielded products with a high efficiency of 92%. The study highlighted that the catalyst is effective for DHP synthesis and suitable for scaling up. It was found that 85°C is the optimal reaction temperature, as temperatures above 85°C resulted in decreased yields due to decomposition. The method offers several advantages, including the use of a recyclable catalyst, a simple procedure, easy workup, and high product yields (Scheme **11**) [43].

**Scheme 12:**
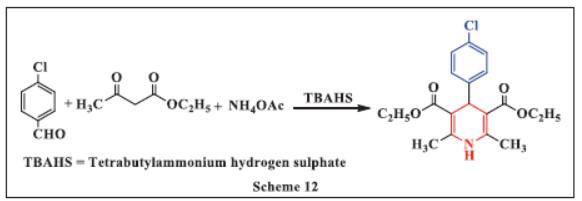
Multi-component one-pot synthesis of 1,4 dihydropyridines using Tetrabutylammonium hydrogen sulphate catalyst.

**Vijender *et al.,*** in **2018,** presented an efficient and versatile method for synthesizing 1,4-DHPs under mild reaction conditions. The process involved the condensation of ethyl acetoacetate, ammonium acetate, and substituted aryl aldehydes with tetra-n-butylammonium hydrogen sulfate (TBAHS) at 70°C in solvent-free conditions. TBAHS, a well-known heterogeneous solid catalyst, is prized for its selectivity, inertness, and thermal stability, making it highly effective in organic synthesis. The method offers several advantages, including improved yields, faster reaction times, a simpler work-up procedure, easy catalyst isolation, and the ability to reuse the catalyst across multiple reaction cycles (Scheme **12**) [44].

**Scheme 13:**
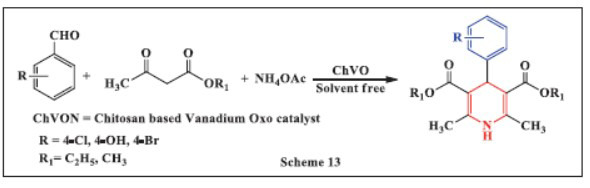
Multi-component one-pot synthesis of 1,4 dihydropyridines using Chitosan based vanadium oxo catalyst.

**Safaiee *et al.,*** in **2018,** reported a simple and efficient one-pot synthesis of 1,4-dihydropyridines (1,4-DHPs) using a chitosan-based vanadium oxo catalyst (ChVO) under solvent-free conditions. The reaction involved mixing aldehydes, β-keto-esters, and ammonium acetate, with ChVO added at 85 °C for an appropriate duration. TLC was employed to monitor the progress of the reaction. The ChVO catalyst enabled excellent product yields in a solvent-free environment. The process is straightforward, clean, and highly effective, offering several advantages for the synthesis of DHPs, including solvent-free conditions, short reaction times, easy availability, catalyst recyclability, low cost, and the simplicity and stability of the catalyst (Scheme **13**) [45].

**Scheme 14:**
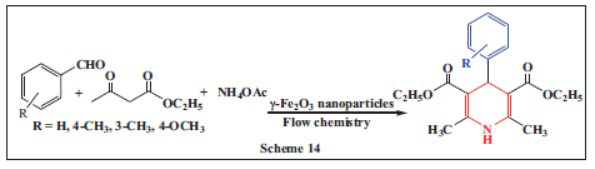
Multi-component one-pot synthesis of 1,4 dihydropyridines using Maghemite (γ-Fe_2_O_3_) nanoparticles using flow chemistry.

**Wei *et al.,*** in **2018,** introduced a novel micro-flow system for the continuous synthesis of 1,4-DHPs under solvent-free conditions *via* the Hantzsch reaction, using microwave irradiation. The system utilized γ-Fe_2_O_3_ nanoparticles as a catalyst and featured a valve-assisted micromixer for continuous precipitation of γ-Fe_2_O_3_. An activated molecular sieve was employed to absorb water, facilitating the completion of the reaction and enhancing conversion. The condensation reaction, involving ammonium acetate, ethyl acetoacetate, and aldehydes, was initially conducted for 8 minutes at 40 °C, yielding 89.0% of the enaminoester. Higher temperatures further improved the reaction efficiency. The micro-flow system enhances yield and selectivity by providing shorter diffusion pathways and improved mass transfer rates. It also offers higher safety through real-time, small-scale reactions. This method has found widespread use in organic chemistry, analytical chemistry, and biotechnology. Compared to traditional heating, microwave chemistry accelerates reactions, reduces side products, and improves product purity. The combination of flow chemistry and microwave irradiation further promotes the reaction. When comparing different heating methods in a packed bed flow reactor, microwave irradiation demonstrated superior reaction efficiency over air heating and oil-bath heating. Additionally, γ-Fe_2_O_3_ could be recycled multiple times without significant loss of catalytic activity (Scheme **14**) [46].

**Scheme 15:**
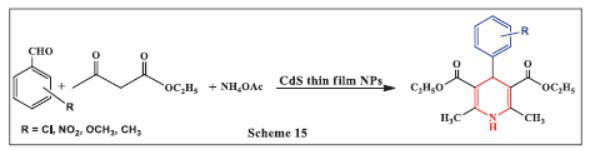
Multi-component one-pot synthesis of 1,4 dihydropyridines using Cadmium sulphide thin film nanoparticles.

**Lavanya *et al.,*** in **2019,** developed an effective one-pot, three-component synthesis of Hantzsch 1,4-DHPs with high yields using recyclable nanocrystalline CdS thin film technology. The CdS thin film catalyst was synthesized *via* a chemical bath deposition method. The reaction, which involved a mixture of aromatic aldehydes, ethyl acetoacetate, ammonium acetate, and nanocrystalline CdS thin film, was refluxed at 75-80°C. The study also examined the effects of temperature, substituents, and mole ratios on the reaction. The heterogeneous catalyst could be easily recovered from the reaction mixture and reused at least five times without losing its catalytic activity. This approach offers several advantages, including simple experimental procedures, easy isolation, clean reactions, short reaction times, and mild conditions with no byproducts. The method’s simplicity and efficiency make it a promising technology for a variety of other organic processes (Scheme **15**) [47].

**Scheme 16:**
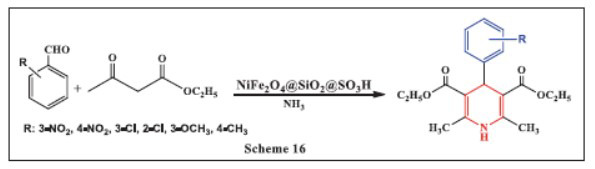
Multi-component one-pot synthesis of 1,4 dihydropyridines using Nickel Ferrite, silicon dioxide and sulfonic acid catalyst.

**Zeynizadeh *et al.,*** in **2019,** explored the catalytic activity of a Ni-nanocomposite in the Hantzsch synthesis of 1,4-DHPs through a one-pot condensation reaction. This reaction utilized aqueous ammonia, aromatic aldehydes, and 1,3-diketones (such as ethyl acetoacetate or 4-hydroxycoumarin) in water at 70°C, with water serving as a green solvent. The reactions were completed in less than 10 to 100 minutes, resulting in high to excellent yields. The method offers multiple advantages, including mild reaction conditions, the use of water as an environmentally friendly solvent, the stability and easy separation of the magnetic Ni-nanocatalyst, high product yields, broad substrate tolerance, and excellent catalyst reusability. These benefits highlight the method’s potential for sustainable organic synthesis (Scheme **16**) [48].

**Scheme 17:**
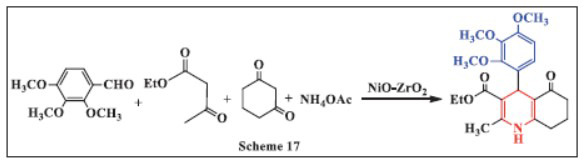
Multi-component one-pot synthesis of 1,4 dihydropyridines using Nickel oxide loaded on zirconia catalyst.

**Sandeep *et al.,*** in **2019,** reported a method for rapidly synthesizing 18 unsymmetrical 1,4-dihydropyridine compounds using nickel oxide loaded on zirconia (NiO/ZrO_2_). The synthesis of various 1,4-DHPs was carried out using ethanol (EtOH) as the solvent. NiO/ZrO_2_ was added to a mixture of equimolar amounts of substituted aldehydes, ethyl acetoacetate, ammonium acetate, and 1,3-cyclohexadione, and the reaction was stirred at room temperature. The Lewis acid properties of the catalyst made it an excellent choice for this one-pot, four-component reaction, yielding products in high yields of 89-98% within 20-45 minutes. Notably, the catalyst could be reused up to six times. This green, cost-effective approach benefits from the use of ethanol as a solvent and enables the reaction to proceed at ambient temperature, offering a sustainable and efficient method for synthesizing 1,4-DHPs (Scheme **17**) [49].

**Scheme 18:**
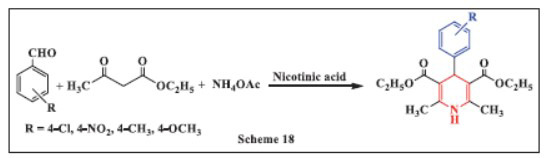
Multi-component one-pot synthesis of 1,4 dihydropyridines using Nicotinic acid catalyst.

**Jamal *et al.,*** in **2019,** reported the synthesis of 1,4-DHPs using nicotinic acid as a catalyst. The reaction involved heating a mixture of benzaldehyde, ethyl acetoacetate, and ammonium acetate in the presence of various amounts of catalyst. The effectiveness of the catalyst was evaluated across different solvents and temperatures. By using 0.1 g of the catalyst at 80°C under solvent-free conditions, excellent yields of the coupling products were achieved under optimal conditions. This method offers several advantages, including ease of handling, high yield, rapid reaction times, a simple setup, the use of a reusable and low-cost catalyst, and the ability to conduct multicomponent reactions without the need for solvents, making it a promising approach for sustainable organic synthesis (Scheme **18**) [50].

**Scheme 19:**
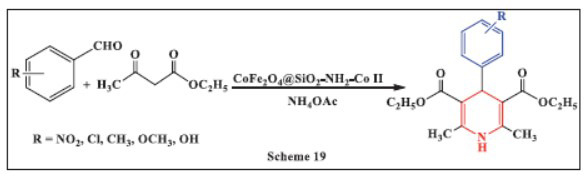
Multi-component one-pot synthesis of 1,4 dihydropyridines using Amino-functionalized cobalt ferrite-silica composite with cobalt(II) coordination catalyst.

**Allahresani *et al.,*** in **2020,** reported a simple, green, one-pot method for synthesizing functionalized 1,4-dihydropyridines (1,4-DHPs) catalyzed by CoFe_2_O_4_@SiO_2_-NH_2_-Co(II) nanoparticles. The synthesis involved reacting aldehydes, ethyl acetoacetate, and ammonium acetate in the presence of this magnetic nanocatalyst in a 1:1 mixture of ethanol and water. The 1,4-DHP compounds were obtained in good to excellent yields (60-96%) using refluxing with a green solvent. This method offers several advantages, including rapid purification, high atom efficiency, short reaction times, the use of an environmentally friendly solvent, excellent yields, and a simple work-up procedure. The approach holds significant promise for broader applications in drug discovery and chemical synthesis (Scheme **19**) [51].

**Scheme 20:**
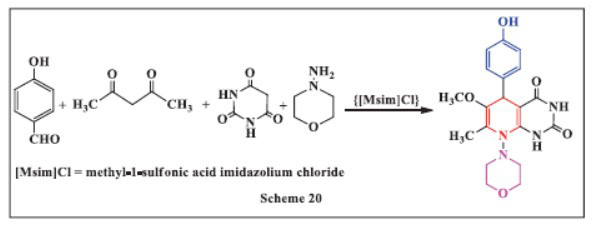
Multi-component one-pot synthesis of 1,4 dihydropyridines using Methyl-1-sulfonic acid imidazolium chloride catalyst.

**Ahmed *et al.,*** in **2020,** developed an efficient one-step method for synthesizing new Hantzsch 1,4-DHPs with high yields through a four-component reaction in ethanol. The reaction involved 4-hydroxybenzaldehyde, acetylacetone, various primary amines, and barbituric acid in the presence of 3-methyl-1-sulfonic acid imidazolium chloride {[Msim]Cl} as an acidic ionic liquid. The use of {[Msim]Cl} as a catalyst enhanced the multicomponent reaction by providing more effective and environmentally friendly catalysis. The method offers several advantages, including a straightforward synthesis process, short reaction times, high product yields, low chemical costs, reduced pollution, and minimized use of hazardous solvents. (Scheme **20**) [28].

**Scheme 21:**
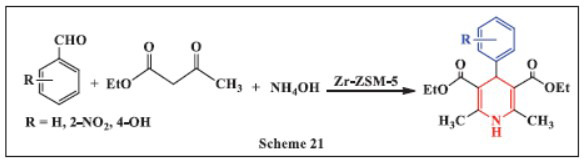
Multi-component one-pot synthesis of 1,4 dihydropyridines using Zeolite catalyst.

**Uppalaiah *et al.,*** in **2020,** introduced an innovative and efficient method for synthesizing 1,4-DHPs by reacting aceto-acetanilide or ethyl acetoacetate, ammonium acetate (AcONH_4_) or ammonium hydroxide (NH_4_OH), and various aromatic aldehydes in the presence of a highly efficient heterogeneous solid catalyst. The reaction mixture, consisting of aceto-acetanilide or ethyl acetoacetate, distilled benzaldehyde, and AcONH_4_/NH_4_OH, was dissolved in ethanol and added to Zr-ZSM-5, then heated under reflux conditions. The reactions achieved excellent product yields (87-95%) within 27-35 minutes at the optimal temperature in ethanol. The catalyst was easily recycled four to five times without a noticeable loss in catalytic activity. This method is simpler and more efficient than other approaches due to the use of a non-toxic, recyclable, reusable, affordable, and environmentally friendly catalyst (Scheme **21**) [52].

**Scheme 22:**
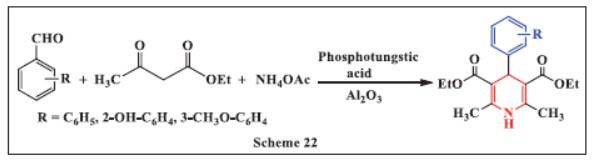
Multi-component one-pot synthesis of 1,4 dihydropyridines using Phosphotungstic acid, aluminium oxide catalyst.

**Bosica *et al.,*** in **2020,** reported the synthesis of 1,4-DHPs using heterogenized phosphotungstic acid supported on alumina (40%). The use of green solvents, such as water or ethanol, was found to negatively impact the reaction while increasing the temperature had little effect on yield or reaction time. After optimizing the reaction conditions and screening various heterogeneous catalysts, high yields of over 75% were achieved within two to three hours. The selected catalyst was shown to be reusable for up to 8 consecutive cycles without significant loss of activity, passing the heterogeneity test. Notably, when aliphatic aldehydes were used, the traditional 1,4-DHPs were produced, whereas aromatic aldehydes led to the previously reported regioisomer (Scheme **22**) [53].

**Scheme 23:**
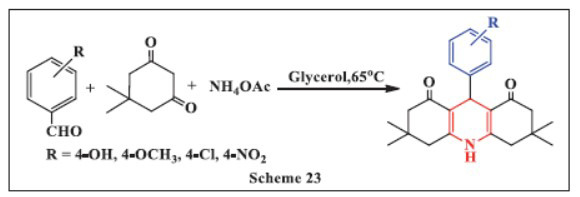
Multi-component one-pot synthesis of 1,4 dihydropyridines using Glycerol as catalyst.

**Tiwari *et al.,*** in **2020**, described an eco-friendly, simple, and clean one-pot, multi-component Hantzsch reaction using glycerol as a promoting medium for the synthesis of 1,4-DHPs. Glycerol was found to have favorable properties, acting as both a solvent to dissolve substrate molecules and an activator of electrophilic substrate molecules due to its strong hydrogen bonding capabilities. The reaction involved mixing aromatic aldehydes, dimedone, and ammonium acetate with glycerol and then heating the mixture at 65 °C for a specified duration. The method, conducted under metal-free mild conditions, offers several advantages, including faster reaction times, cost-effectiveness, and environmental friendliness, while providing moderate to excellent yields and good atom economy. This approach presents a promising, sustainable route for synthesizing physiologically relevant 1,4-DHPs (Scheme **23**) [54].

**Scheme 24:**
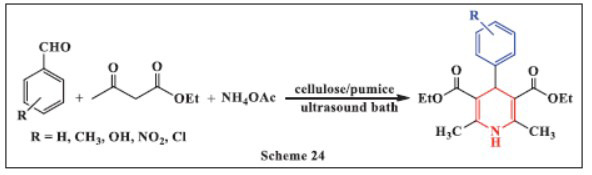
Multi-component one-pot synthesis of 1,4 dihydropyridines using Cellulose/pumice catalyst.

**Valadi *et al.,*** in **2020,** reported the ultrasound-assisted synthesis of 1,4-DHPs using an efficient cellulose/pumice nanocatalyst. The reaction involved mixing aldehyde derivatives, ethyl acetoacetate, and ammonium acetate at 80°C in the presence of the cellulose/pumice nanocatalyst in ethanol as a polar solvent to enhance the reaction. The results demonstrated that the combination of the co-catalytic effect of the novel green cellulose/pumice nanocatalyst and ultrasonication led to high reaction yields (97%) in a short reaction time (10 minutes) under mild conditions. This catalytic system showed excellent recyclability and reusability while maintaining effective catalytic activity. The approach offers a promising strategy for utilizing natural catalytic supports in green and efficient chemical synthesis (Scheme **24**) [55].

**Scheme 25:**
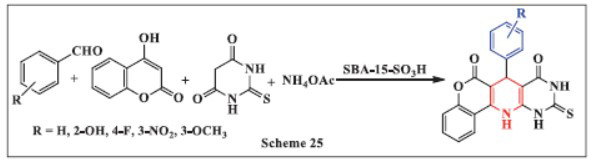
Multi-component one-pot synthesis of 1,4 dihydropyridines using Santa Barbara Amorphous-15 catalyst.

**Sayahi *et al.,*** in **2021,** described a one-pot synthesis of novel 1,4-DHPs using SBA-15-SO_3_H as the catalyst. The reaction was conducted by heating a mixture of substituted benzaldehyde, 4-hydroxycoumarin, thiobarbituric acid, and ammonium acetate at 120 °C for 5 hours without the use of any solvent. The catalyst demonstrated excellent reusability, as it could be recycled for up to four consecutive reactions without compromising the final yield. Advantages of this method include the ability to produce products in relatively short reaction times, the use of easily accessible starting materials, and high product yields, making it an efficient and practical approach for the synthesis of 1,4-DHPs (Scheme **25**) [56].

**Scheme 26:**
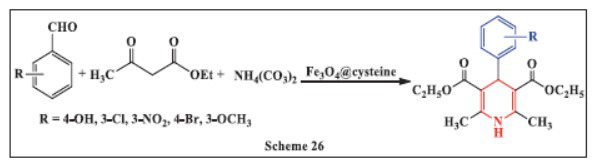
Multi-component one-pot synthesis of 1,4 dihydropyridines using Cysteine-functionalized magnetite nanoparticles catalyst.

**Ghosh *et al.,*** in **2021,** reported the use of Fe_3_O_4_@cysteine magnetic nanoparticles as a catalyst for a three-component, one-pot reaction between aldehydes, ethyl acetoacetate, and ammonium carbonate to synthesize 1,4-DHPs. This method offers several advantages, including good to high yields (79-96%), short reaction times, simple work-up procedures, and mild reaction conditions. The approach also avoids the use of hazardous organic solvents, generates minimal waste, and eliminates the need for chromatographic purification. The Fe_3_O_4_@cysteine nanoparticles can be easily recovered magnetically and reused for multiple runs, making the process environmentally friendly and cost-effective. The catalyst is highly effective at lower concentrations, and the method successfully works on a gram scale, offering a sustainable and efficient route for the production of 1,4-DHPs (Scheme **26**) [57].

**Scheme 27:**
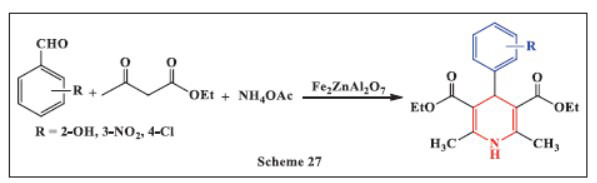
Multi-component one-pot synthesis of 1,4 dihydropyridines using Diiron(III) zinc dialuminum heptaoxide catalyst.

**Zargarzadeh *et al.,*** in **2021,** reported the use of Fe_2_ZnAl_2_O_7_ as a catalyst in a three-component reaction between ammonium acetate, ethyl acetoacetate, and aromatic aldehydes, creating a simple, affordable, and efficient one-pot approach for synthesizing 1,4-DHPs with high yields. The reaction was performed at 70-80 °C under solvent-free conditions. The method offers several advantages, including short reaction times, high to exceptional product yields, clean and safe reaction conditions, and the use of a potent new heterogeneous catalyst. The developed catalyst demonstrated remarkable efficiency and reusability, as it could be recycled for four to five runs without a significant decrease in yield or catalytic performance, making this approach both sustainable and cost-effective for the synthesis of 1,4-DHPs (Scheme **27**) [58].

**Scheme 28:**
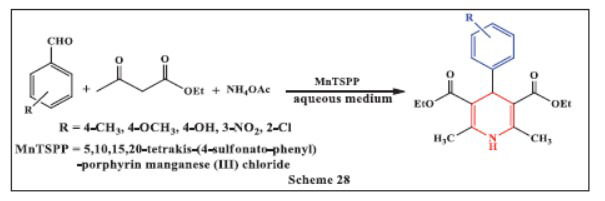
Multi-component one-pot synthesis of 1,4 dihydropyridines using 5,10,15,20-tetrakis-(4-sulfonato-phenyl)-porphyrin manganese (III) chloride catalyst.

**Remaily *et al.,*** in **2021,** reported the facile and highly efficient one-pot synthesis of 1,4-DHPs using MnTSPP (5,10,15,20-tetrakis-(4-sulfonato-phenyl)-porphyrin manganese (III) chloride) as a catalyst. The method involved the reaction of aromatic aldehydes, ethyl acetoacetate, and ammonium acetate in the presence of MnTSPP under mild conditions. The reactions were carried out in a mixture of ethanol and water for 15-80 minutes, yielding products with high to exceptional yields. This protocol offers several notable advantages, including the avoidance of hazardous organic solvents, simple experimental procedures, high product yields, and the availability of a commercially accessible catalyst. Additionally, the method is characterized by short reaction times, straightforward work-up procedures, low-cost and non-toxic catalysts, broad substrate compatibility, and the catalyst's excellent reusability, making it both cost-effective and environmentally friendly (Scheme **28**) [59].

**Scheme 29:**
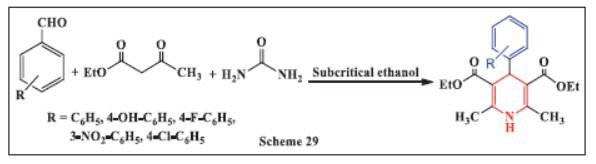
Multi-component one-pot synthesis of 1,4 dihydropyridines using Subcritical ethanol catalyst.

**Ersatir *et al.,*** in **2022,** developed a simple, eco-friendly, and highly efficient method for the synthesis of 1,4-DHPs. This method employs a one-pot MCR in sub-critical ethanol to convert a variety of aromatic aldehydes, ethyl acetoacetate, and urea into the corresponding 1,4-DHPs. The reaction was conducted at 220°C for 60 minutes, with progress monitored by TLC. The method offers several advantages, including good product yields, quick reaction times, and straightforward work-up conditions, making it a highly efficient and sustainable approach for synthesizing 1,4-DHPs (Scheme **29**) [60].

**Scheme 30:**
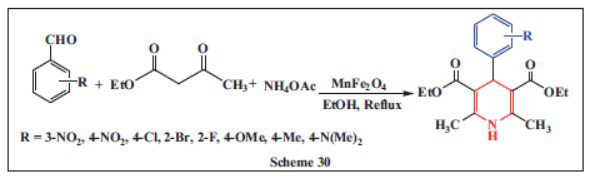
Multi-component one-pot synthesis of 1,4 dihydropyridines using Manganese ferrite catalyst.

**Moradi *et al.,*** in **2022,** described an effective one-pot synthesis of various 1,4-DHPs. They reported the condensation reaction of several aldehyde derivatives, ethyl acetoacetate, and ammonium acetate in the presence of superparamagnetic manganese ferrite nanoparticles (MnFe_2_O_4_) at 80°C. The superparamagnetism of MnFe_2_O_4_ is highly beneficial as they become magnetized in the presence of an external magnetic field but lose magnetization when the field is removed. This property allows the particles to disperse well in the reaction medium, facilitating the rapid interaction of reactants with the nanoparticle surface, and making them efficient catalysts. The method offers several advantages, including high selectivity, excellent product purity, good yields, quick reaction times, and environmentally friendly conditions. Additionally, the process is simple to carry out, and the catalyst can be easily extracted and reused multiple times without significantly reducing the yield of the final product, making it an efficient and sustainable approach for synthesizing 1,4-DHPs (Scheme **30**) [61].

**Scheme 31:**
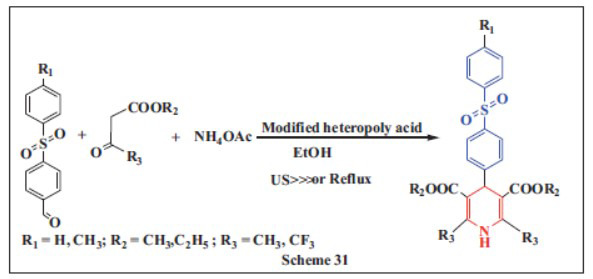
Multi-component one-pot synthesis of 1,4 dihydropyridines using Modified heteropoly acid catalyst.

**Mokhtar *et al.,*** in **2022,** reported the use of modified heteropoly acid (HPA) catalysts, specifically 5 wt% WO_X_-FeP and 5 wt% MoO_X_-FeP, in a one-pot synthesis approach under ultrasonic irradiation to create novel Hantzsch 1,4-DHPs with a diphenyl sulfone moiety. The method is straightforward, environmentally friendly, and allows for the synthesis of a wide range of 1,4-DHP derivatives. The reaction achieves excellent product yields (88-92%) with the use of HPA catalysts. FeP-supported HPA catalysts demonstrated enhanced reducibility and acidity. The 5W-FeP catalyst exhibited superior catalytic efficiency in the Hantzsch reaction. The catalysts exhibited improved catalytic performance due to enhanced Lewis acid site density, redox properties, and surface area, outperforming the parent phosphomolybdic and phosphotungstic acids. Additionally, these catalysts can be easily separated from the reaction system and reused up to six times with minimal loss of activity, making the process both sustainable and cost-effective for the production of 1,4-DHPs (Scheme **31**) [62].

**Scheme 32:**
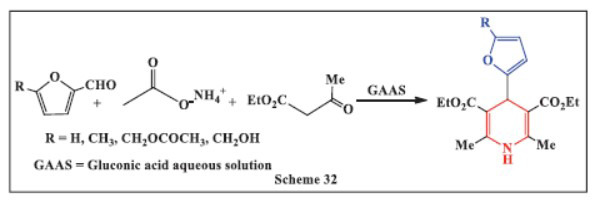
Multi-component one-pot synthesis of 1,4 dihydropyridines using Gluconic acid aqueous solution as catalyst.

**Anchan *et al.,*** in **2023,** described the one-pot, multicomponent synthesis of 1,4-DHPs using carbohydrate-derived 5-substituted-2-furaldehydes and gluconic acid aqueous solution (GAAS) as the green organocatalyst. Motivated by the numerous advantages of organocatalysts-such as insensitivity to moisture and oxygen, accessibility, affordability, and low toxicity-the researchers employed GAAS as an environmentally friendly catalyst for the synthesis of 1,4-DHPs. The reactions were completed in six hours at 60 °C with an equivalent or slight excess of reagents. To demonstrate the sustainability of the proposed methodology, 10 g of furfural was subjected to the optimal conditions. The results showed that GAAS, an effective, cheap, recyclable, non-toxic, and renewable catalyst, facilitated the reaction efficiently. Following the general synthetic protocol under optimal conditions (60 °C, 3-6 hours, and 25 mol% GAAS catalyst), good to excellent isolated yields of various 1,4-DHP derivatives were achieved, proving the method to be both efficient and environmentally sustainable (Scheme **32**) [63, 64].

**Scheme 33:**
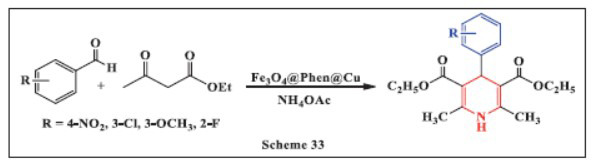
Multi-component one-pot synthesis of 1,4 dihydropyridines using Copper-functionalized 1,10-Phenanthroline-coated magnetite (Fe_3_O_4_) nanocomposite catalyst.

**Willong *et al.,*** in **2023,** described recent advancements in the synthesis and aromatization of 1,4-DHPs using a magnetic catalyst, Fe_3_O_4_@Phen@Cu. The team conducted a one-pot reaction of benzaldehyde, ethyl acetoacetate, and ammonium acetate in the presence of a PEG solvent at 60°C to evaluate the catalytic activity of Fe_3_O_4_@Phen@Cu and determine the optimal reaction conditions for synthesizing 1,4-DHPs. The reaction provided high yields (86-97%) in a short time, showcasing the efficiency of Fe_3_O_4_@Phen@Cu as an effective, eco-friendly catalyst. The process offers several advantages, including high product yield, rapid reaction times, a cost-effective catalyst, simple catalyst preparation, easy product separation, and catalyst accessibility. Additionally, performing the reaction in an aqueous solvent aligns with green chemistry principles, further enhancing the sustainability of the approach (Scheme **33**) [65].

**Scheme 34:**
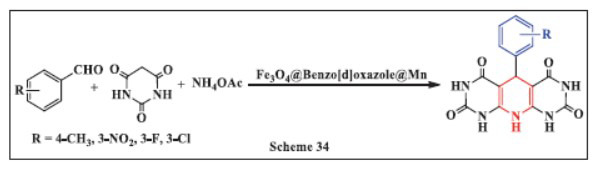
Multi-component one-pot synthesis 1,4 dihydropyridines using Manganese-functionalized Benzo[d]oxazole-coated magnetite (Fe_3_O_4_) nanocomposite catalyst.

**Ichie *et al.,*** in **2023,** described the synthesis of 1,4-DHPs from barbituric acid using Fe_3_O_4_@Benzo[d]oxazole@Mn as a catalyst. The one-pot reaction, involving benzaldehyde, barbituric acid, and ammonium acetate, was carried out in ethanol at 75°C. The study found this reaction to be the most effective for producing 1,4-DHPs, with the reaction efficiency diminishing in the presence of non-polar solvents. The magnetic catalyst used in the reaction offered several advantages, including ease of preparation, low toxicity, sustainability of reaction conditions, quick reaction times, and excellent recyclability, making it an ideal choice for efficient and environmentally friendly synthesis (Scheme **34**) [66].

**Scheme 35:**
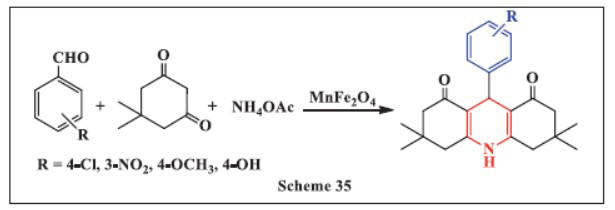
Multi-component one-pot synthesis of 1,4 dihydropyridines using Manganese ferrite catalyst.

**Zhang *et al.,*** in **2023,** introduced an effective method for synthesizing 1,4-DHPs using the MnFe_2_O_4_ catalyst. The multicomponent reaction was conducted by stirring benzaldehyde, dimedone, and ammonium acetate in the presence of MnFe_2_O_4_. TLC was employed to monitor the reaction's progress. It was observed that longer reaction times were required when substituted aldehydes were in the *ortho* position. The method yielded the desired products in a relatively short period, with good to excellent yields ranging from 88-98%. Additionally, the magnetic MnFe_2_O_4_ nanocatalyst maintained its catalytic activity over four reuse cycles. It was easily removed from the reaction system using a magnet, offering a simple and efficient solution for the synthesis of 1,4-DHPs (Scheme **35**) [67].

**Scheme 36:**
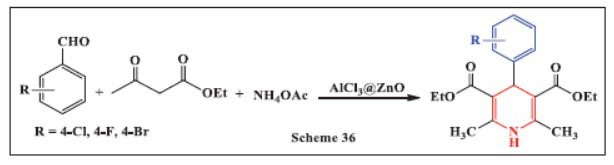
Multi-component one-pot synthesis of 1,4 dihydropyridines using Aluminum Chloride-functionalized Zinc oxide nanocomposite catalyst.

**Shinde *et al.,*** in **2023,** reported a one-pot, solvent-free method for the synthesis of 1,4-DHPs using a nanocrystalline AlCl_3_@ZnO nanocatalyst. This catalyst was developed by loading 20% AlCl_3_ onto ZnO nanoparticles through a simple wet-impregnation method. The AlCl_3_@ZnO nanocrystalline catalyst demonstrated high effectiveness in catalyzing the Hantzsch dihydropyridine reactions using a variety of aromatic aldehydes, ethyl acetoacetate, and ammonium acetate. The catalyst, with an average particle size of 70-80 nm, provided a 92% yield for the synthesis of 1,4-DHPs under solvent-free and room-temperature conditions. This catalyst offers significant advantages over traditional homogeneous and heterogeneous catalysts due to its high efficiency, recyclability, and environmental friendliness, positioning it as a promising alternative in catalytic reactions (Scheme **36**) [68].

**Scheme 37:**
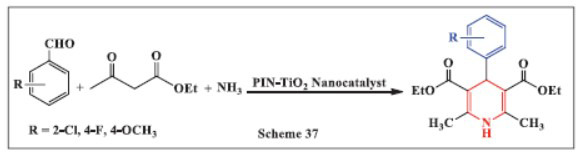
Multi-component one-pot synthesis of 1,4 dihydropyridines using Polyindole-functionalized titanium dioxide nanocomposite catalyst.

**Phasage *et al.,*** in **2024,** described a one-pot, green synthesis method for 1,4-DHPs using a polyindole-TiO_2_ nanocatalyst under solvent-free conditions. This study utilized the combination of polyindole and TiO_2_ nanoparticles as an efficient heterogeneous catalyst for a multi-component Hantzsch reaction. The reaction involved different aromatic aldehydes, methyl acetoacetate, and aqueous ammonium to synthesize 1,4-DHPs at ambient temperature without the need for a solvent. A wide range of aldehydes and methyl acetoacetates, including heteroaromatic and polyaromatic compounds, showed high functional group tolerance, yielding the desired products with medicinally relevant moieties. The method offers several advantages, such as the use of a low-cost, stable, recyclable, and safe catalyst, faster reaction times, higher yields, and simple product isolation, making it a promising alternative to current protocols for synthesizing 1,4-DHPs (Scheme **37**) [69].

**Scheme 38:**
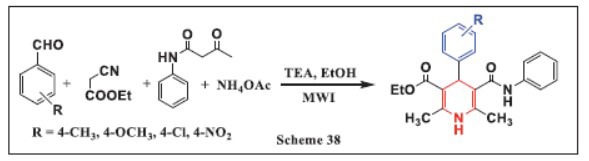
Multi-component one-pot synthesis of 1,4 dihydropyridines using Triethylamine (TEA) catalyst.

**Pachipulusu *et al.,*** in **2024,** developed an eco-friendly method for synthesizing 1,4-DHPs using microwave irradiation. This process uses ethanol as a green solvent and triethylamine as a catalyst, facilitating fast reactions (under 30 minutes) with high yields (90%-96%). The method is energy-efficient and eliminates the need for column chromatography, simplifying the purification process. The reaction is a four-component process, which reduces the number of reagents and solvents needed. The products were characterized using various spectroscopic techniques. This approach offers several advantages, such as increased atom economy, faster production, and simplicity, making it particularly valuable in pharmaceutical research for generating a wide variety of heterocyclic compounds important for drug development (Scheme **38**) [70].

**Scheme 39:**
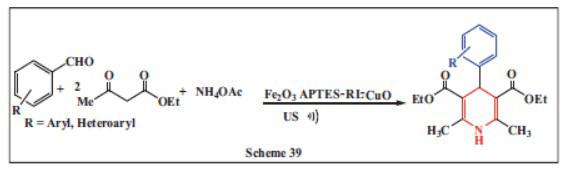
Multi-component one-pot synthesis of 1,4 dihydropyridines using Copper oxide (CuO) functionalized with (3-aminopropyl)triethoxysilane (APTES) and Rhodamine B (RL) on Magnetite (Fe_3_O_4_) Nanocomposite catalyst.

**Dolatyari *et al.,*** in **2024,** reported an eco-friendly method for synthesizing 1,4-DHPs using a novel catalyst made from biocompatible D-(+)-ribonic γ-lactone (RL) combined with Fe_3_O_4_ and CuO nanoparticles (CuO NPs). The RL acted as a support for CuO NPs, enhancing their catalytic activity by preventing agglomeration and improving their stability. This catalyst, Fe_3_O_4_-APTES-RL-CuO, was used in the one-pot synthesis of 1,4-DHPs under ultrasound (US) conditions, achieving a high yield of 95% in about 15 minutes. The catalyst was easily extracted using an external magnet and maintained its catalytic activity after five reuses without degradation. The catalyst’s structure and properties were thoroughly characterized using FT-IR, XRD, FE-SEM, EDS, TGA, and VSM, demonstrating its potential as an effective, environmentally friendly catalyst for the synthesis of 1,4-DHPs (Scheme **39**) [71].

**Scheme 40:**
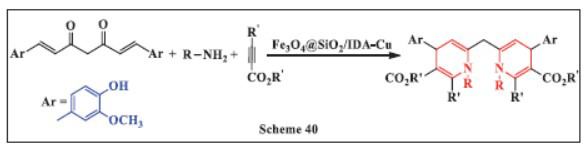
Multi-component one-pot synthesis of 1,4 dihydropyridines using Silica-coated magnetic nanoparticles functionalized with iminodiacetic acid-copper catalyst.

**Motamed *et al.,*** in **2024,** introduced a green method for synthesizing 1,4-DHPs using a novel catalytic system consisting of silica-coated magnetic nanoparticles functionalized with iminodiacetic acid-copper (Fe_3_O_4_@SiO_2_/IDA-Cu). The one-pot reaction involved curcumin, primary amines, and activated acetylenic compounds and was carried out in an aqueous medium at room temperature. This approach offered several notable benefits, including high yields of the desired 1,4-DHP compounds, easy separation of the products and catalyst, and the reusability of the catalyst. The use of this eco-friendly, sustainable catalyst not only improved the efficiency of the reaction but also contributed to promoting greener chemical synthesis practices (Scheme **40**) [72].

**Scheme 41:**
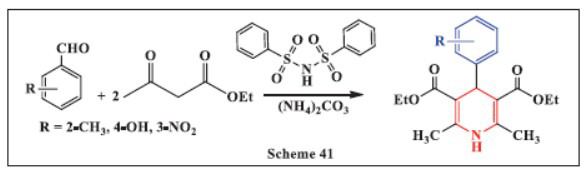
Multi-component one-pot synthesis of 1,4 dihydropyridines using *N*-(phenylsulfonyl)benzene sulfonamide catalyst.

**Ghoderao *et al.,*** in **2024,** investigated the synthesis of 1,4-DHPs using *N*-(phenylsulfonyl)benzene sulfonamide as an organocatalyst in an environmentally benign solvent at 75°C. This method is straightforward, cost-effective, and efficient, offering excellent yields, fast reaction times, simple workup, and catalyst recycling and reusability. These features contribute to both economic and ecological advantages. The synthesis was performed with various functional groups, demonstrating its versatility and compatibility. The product was separated by adding water to the reaction mixture, followed by filtration and washing. The catalyst's recyclability further enhances the sustainability of the process. The study also explored the impact of different solvents on the reaction rate and yield. It was observed that no product formed when the reaction was carried out in THF, and only trace amounts of product were obtained when acetonitrile was used. However, the solvent-free reaction and the use of an EtOH:H_2_O mixture provided excellent yields, highlighting this composition as an eco-friendly and cost-effective solvent system for the synthesis of 1,4-DHP derivatives (Scheme **41**) [73].

**Scheme 42:**
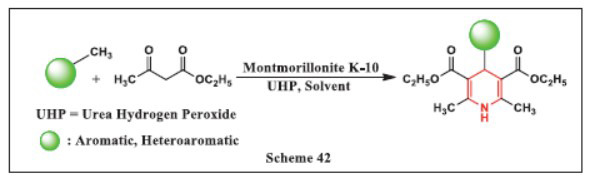
Multi-component one-pot synthesis of 1,4 dihydropyridines using Montmorillonite K-10 catalyst.

**Singh *et al.,*** in **2024,** introduced a rapid, efficient, and eco-friendly method for synthesizing a variety of Hantzsch 1,4-DHPs using montmorillonite K-10 as a catalyst under solvent-free conditions. This method involves reacting methyl arynes, which are sustainable substitutes for aryl aldehydes, with active methylene compounds and urea hydrogen peroxide (UHP), which acts as both an oxidizing agent and a source of ammonia. The reaction is accelerated using microwave irradiation, significantly improving the efficiency of the synthesis process. Key advantages of this approach include the elimination of solvents, which not only reduces waste generation but also shortens reaction times and lowers energy costs. The methodology is designed for simplicity, featuring a straightforward setup and work-up process, and provides high yields of the desired products. Additionally, the montmorillonite K-10 catalyst is highly effective and can be recycled multiple times without significant loss of activity, contributing to the sustainability and cost-effectiveness of the process (Scheme **42**) [74].

**Scheme 43:**
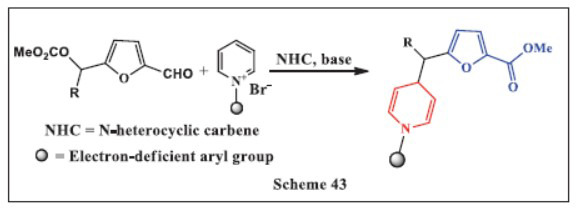
Multi-component one-pot synthesis of 1,4 dihydropyridines using *N*-heterocyclic carbene catalyst.

**Xu *et al.,*** in **2024,** introduced a novel method for synthesizing 4-(furan-2-ylmethyl)-1,4-DHPs through N-heterocyclic carbene (NHC) catalysis, utilizing pyridinium salts and furfural derivatives. This innovative approach involves the formation of NHC-bound trienolates from the furfural derivatives, which are then added to the C-4 position of *N*-aryl pyridinium salts. The reaction is noted for its impressive yields, ranging from 41% to 99%, and it achieves significant regioselectivity, with a ratio exceeding 20:1 in all cases. This protocol not only showcases the versatility of NHC catalysis in constructing complex molecular architectures but also demonstrates its potential application in pharmaceutical synthesis, where both furan and dihydropyridine motifs are crucial for developing bioactive compounds. This method provides an efficient strategy for the selective synthesis of functionalized 1,4-DHPs, which can be of significant value in medicinal chemistry (Scheme **43**) [75].

## CONCLUSION

1,4-DHPs are highly regarded for their broad biological and pharmacological properties, which make them a key focus in the field of synthetic organic chemistry. As research in this area continues to evolve, it is expected that new derivatives of 1,4-DHP will be discovered, broadening their potential applications in clinical settings. The development of efficient and sustainable methods for synthesizing these compounds has been significantly advanced through the use of catalyst-driven MCRs. Recent innovations in catalyst-driven MCRs have led to substantial improvements in several important areas, such as yields and selectivity, reduced reaction times and energy consumption, broader substrate scope and environmental sustainability. These advancements have contributed to reducing waste and minimizing the environmental impact, aligning with the growing emphasis on green chemistry in modern chemical synthesis. These developments have profound implications across various scientific disciplines, particularly in pharmaceutical research and drug discovery. The ongoing evolution of catalyst-driven multicomponent reactions is set to unlock the full potential of 1,4-DHPs, facilitating the development of new therapies, improving the efficiency of chemical synthesis, and fostering technological advancements. As we continue to explore and develop novel catalysts for the synthesis of 1,4-DHP derivatives, future research holds great promise in enabling the creation of new compounds with diverse pharmacological activities. This will not only advance the field of organic synthesis but also contribute to the discovery of next-generation therapeutic agents with broad applications in treating various diseases.

## Figures and Tables

**Fig. (2):**
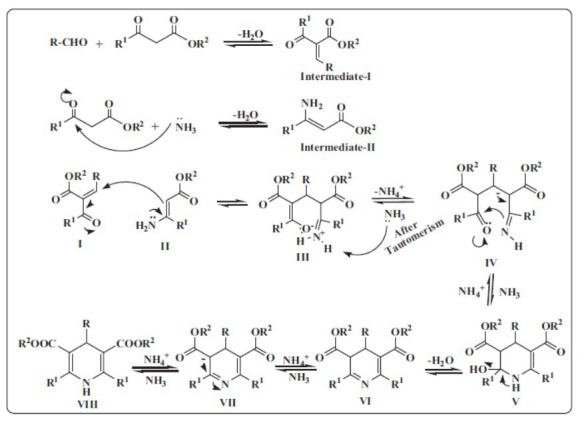
General mechanism of Hantzsch 1,4-DHP.

**Fig. (3):**
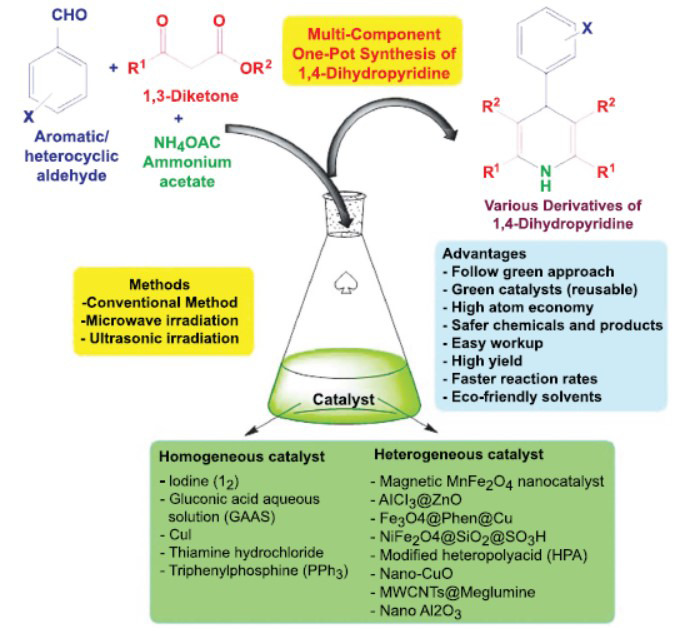
A schematic overview of the multi-component, one-pot synthesis of 1,4-DHP derivatives catalyzed by various catalysts.

## LIST OF ABBREVIATIONS

[Msim]Cl: 3-Methyl-1-sulfonic Acid Imidazolium Chloride
1,2-DHP: 1,2-Dihydropyridine
1,4-DHPs: 1,4-Dihydropyridines
AcONH4: Ammonium Acetate
Al2O3: Aluminum Oxide
AlCl3@ZnO: Aluminum Chloride on Zinc Oxide Nanoparticles
Bi(NO3)3.5H2O: Bismuth Nitrate Pentahydrate
CAN: Ceric Ammonium Nitrate
CdS: Cadmium Sulfide
ChVO: Chitosan-based Vanadium Oxo Catalyst
CoPcS-NH_2_-SiO_2_: Cobalt(II) Sulphonated Phthalocyanine Supported on Aminated Silica
CSCS: Chitosan-Supported Copper (II) Sulfate
CuO NPs: Copper Oxide Nanoparticles
EtOH: Ethanol
Fe2O4@SiO_2_-NH_2_-Co(II): Cobalt(II) Doped Aminated Silica-Coated Iron Oxide Nanoparticles
Fe2ZnAl2O7: Iron Zinc Aluminum Oxide
Fe3O4: Iron(II,III) Oxide (Magnetite)
Fe3O4@Benzo[d]oxazole@Mn: Manganese Functionalized Benzo[d]oxazole-Coated Magnetite Nanoparticles
Fe3O4@cysteine: Cysteine-Coated Magnetite (Iron Oxide) Nanoparticles
Fe3O4@Phen@Cu: Copper-Phenanthroline Functionalized Magnetite Nanoparticles
Fe3O4@SiO2/IDA-Cu: Silica-Coated Magnetite Nanoparticles Functionalized with Iminodiacetic Acid-Copper
Fe3O4-APTES-RL-CuO: Magnetite Functionalized with APTES, Ribonic Lactone, and Copper Oxide
FePcS-NH_2_-SiO_2_: Iron(III) Sulphonated Phthalocyanine Supported on Aminated Silica
GAAS: Gluconic Acid Aqueous Solution
H2O: Water
HPA: Heteropoly Acid
MAS: Microwave-Assisted Synthesis
MCR: Multicomponent Reaction
MnFe2O4: Manganese Ferrite Nanoparticles
MnFe2O4: Manganese Ferrite Nanoparticles
MnTSPP: 5,10,15,20-Tetrakis-(4-sulfonato-phenyl)-porphyrin Manganese (III) Chloride
MoOx-FeP: Molybdenum Oxide-Iron Phosphate (5 wt%)
MWCNTs: Meglumine-Supported Multiwalled Carbon Nanotubes
NADH: Nicotinamide Adenine Dinucleotide (Reduced form)
NADPH: Nicotinamide Adenine Dinucleotide Phosphate (Reduced form)
NH3: Ammonia
NH4+: Ammonium Ion
NH4OH: Ammonium Hydroxide
NHC: N-Heterocyclic Carbene
NiO/ZrO_2_: Nickel Oxide on Zirconia
*n*-WSA: Nano Tungsten Trioxide-Supported Sulphonic Acid
PEG: Polyethylene Glycol
RL: D-(+)-Ribonic γ-Lactone
SBA-15-SO3H: Sulfonic Acid Functionalized Santa Barbara Amorphous-15
TBAHS: Tetra-n-butylammonium Hydrogen Sulfate
*t*-BuOOH: Tert-Butyl Hydroperoxide
THF: Tetrahydrofuran
TiO2: Titanium Dioxide
TiO2: Titanium Dioxide
TLC: Thin Layer Chromatography
UAS: Ultrasound-assisted Synthesis
UHP: Urea Hydrogen Peroxide
V2O5/ZrO2: Vanadium Pentoxide on Zirconium Dioxide
WOx-FeP: Tungsten Oxide-Iron Phosphate (5 wt%)
ZnFe3O4: Zinc Ferrite
Zr-ZSM-5: Zirconium-Doped Zeolite Socony Mobil-5
γ-Fe2O2: γ- Iron(III) Oxide or Maghemite
